# O Efeito da Atorvastatina + Aspirina na Função Endotelial Difere com a Idade em Pacientes com HIV: Um Estudo de Caso-Controle

**DOI:** 10.36660/abc.20190844

**Published:** 2021-06-25

**Authors:** Gerson Gomes dos Santos, Paulo Sérgio Ramos Araújo, Kaliene Maria Estevão Leite, Emmanuelle Tenório Godoi, Adriana Ferraz Vasconcelos, Heloisa Ramos Lacerda

**Affiliations:** 1 Universidade Federal de Pernambuco RecifePE Brasil Universidade Federal de Pernambuco - Pós-graduação em Medicina Tropical, Recife, PE - Brasil; 2 Universidade Federal de Alagoas Instituto de Ciências Farmacêuticas MaceióAL Brasil Universidade Federal de Alagoas - Instituto de Ciências Farmacêuticas, Maceió, AL - Brasil; 3 Instituto de Pesquisa Aggeu Magalhães RecifePE Brasil Instituto de Pesquisa Aggeu Magalhães, Recife, PE - Brasil; 4 Universidade Federal de Pernambuco RecifePE Brasil Universidade Federal de Pernambuco - Medicina Clínica, Recife, PE - Brasil

**Keywords:** HIV, Artérias Carótidas/ultrassonografia, Intima - Média Carotídea, Artéria Braquial, Atorvastatina, Aspirina, Fatores de Risco, Endotélio Vascular/fisiopatologia

## Abstract

**Fundamento:**

Pacientes com HIV têm maior probabilidade de apresentar doenças cardiovasculares quando comparados à população em geral.

**Objetivo:**

Este foi um estudo de caso-controle que teve como objetivo avaliar quais fatores estavam associados a uma redução na espessura médio-intimal da carótida (IMT) da carótida e ao aumento na dilatação mediada por fluxo (DMF) da artéria braquial em pacientes com HIV que receberam atorvastatina + aspirina por um período de 6 meses.

**Métodos:**

Foi realizada uma análise secundária de um ensaio clínico, que incluiu pessoas vivendo com HIV e baixo risco cardiovascular. Um total de 38 pacientes alocados para o braço de intervenção e tratados por 6 meses com uma combinação de atorvastatina + aspirina foram incluídos. Todos os participantes foram submetidos a ultrassonografia da carótida e da artéria braquial, tanto no início quanto no final do estudo. Os casos que responderam com aumento >10% da dilatação braquial (DMF) e redução da espessura médio-intimal da carótida (IMT) foram considerados casos, e aqueles que não responderam foram considerados controles. Avaliamos os fatores associados às respostas positivas obtidas através da IMT e DMF.

**Resultados:**

A redução do IMT não se associou significativamente a nenhum dos fatores de risco avaliados: idade (p = 0,211), sexo (p = 0,260), tabagismo (p = 0,131) ou tempo de diagnóstico do HIV (p = 0,836). Um aumento na DMF foi significativamente associado com a idade entre aqueles na faixa etária de 40-59 anos, p = 0,015 (OR = 4,37; IC 95%: 1,07-17,79).

**Conclusões:**

Os indivíduos mais velhos foram mais propensos a apresentar um aumento na DMF após 6 meses de tratamento com atorvastatina + aspirina.

## Introdução

A expectativa e a qualidade de vida das pessoas infectadas pelo HIV aumentaram significativamente nas últimas décadas. Isso se deve ao grande sucesso da terapia antirretroviral.^1^ Atualmente, viver com o vírus tornou-se uma condição crônica, que impõe o desafio de manter a supressão viral associada ao manejo de comorbidades relacionadas à idade.^2^ Um aumento substancial de óbitos não relacionados à AIDS, como aqueles relacionados às doenças cardiovasculares, têm sido relatados^3^ e são mais prevalentes nesses indivíduos, quando comparados à população em geral.^4 , 5^

Um marcador precoce da aterosclerose é a disfunção endotelial e prevenir essa disfunção pode ser uma alternativa para evitar futuros eventos cardiovasculares. A aspirina e, mais recentemente, as estatinas têm demonstrado efeitos pleiotrópicos, tais como: efeitos imunomoduladores, antitrombogênicos e anti-inflamatórios. Tais medicamentos podem ser uma alternativa para a prevenção primária e secundária desses eventos em pessoas vivendo com HIV.^6 - 8^

Estudos observacionais e de intervenção avaliaram os efeitos das estatinas na melhora da função endotelial e na progressão da espessura da carótida em indivíduos com e sem HIV. Esses estudos utilizaram técnicas de ultrassom não-invasivas, como a dilatação mediada por fluxo (DMF), que mede o fluxo mediado da artéria braquial, e a espessura médio-intimal da carótida (IMT, do inglês *intima-medial thickening* ) e relataram resultados conflitantes.^9 - 12^ Para contribuir com essa discussão, nosso estudo tem como objetivo avaliar os fatores associados à melhora da função endotelial e espessura da carótida medida por DMF e IMT em indivíduos com HIV, com carga viral sob controle, que foram tratados com uma combinação de atorvastatina + aspirina por um período de 6 meses.

## Métodos

Esta foi uma análise secundária de um ensaio clínico ainda não publicado,^13^ no qual foram avaliados 80 participantes que apresentavam baixo risco cardiovascular, medido pelo Escore de Risco Framingham (ERF) e carga viral indetectável.

O estudo foi planejado para 6 meses, utilizando regimes de tratamento com 2 inibidores de transcriptase reversa de nucleosídeos e 1 inibidor não nucleosídeo, que foram randomizados em grupos de intervenção e placebo. Trinta e oito participantes foram alocados para o grupo de intervenção e tratados por 6 meses com uma combinação de 20mg de atorvastatina + 100mg de aspirina, e 42 receberam placebo. O estudo avaliou a eficácia da combinação de drogas através de medidas ultrassonográficas do aumento da dilatação da artéria braquial (DMF), espessura carotídea (IMT) reduzido e marcadores inflamatórios (PCR ultrassensível, ICAM-1, VCAM-1, IL-1, IL -6, TNF-α) e nenhuma diferença foi encontrada entre o grupo de intervenção e o grupo placebo.

No estudo de caso-controle apresentado aqui, foram incluídos 38 indivíduos do grupo intervenção do referido ensaio clínico. O objetivo foi avaliar subgrupos que poderiam se beneficiar do uso de atorvastatina 20mg e aspirina 100mg na redução da aterosclerose subclínica e das doenças cardiovasculares.

Na primeira parte do estudo de caso-controle, um total de 38 indivíduos foram divididos em 24 casos, que foram aqueles que tiveram uma resposta favorável na DMF (≥10% de dilatação da artéria braquial de acordo com o método descrito por Regattieri et al.,^14^ e 14 pacientes considerados controles, por não terem apresentado resposta na DMF.

Na segunda parte do estudo de caso-controle, os 38 indivíduos foram divididos em 29 casos, que eram os indivíduos que apresentaram redução no IMT carotídea, e 9 controles que não apresentaram redução no IMT carotídea.

Todos os indivíduos assinaram o termo de consentimento livre e esclarecido. O estudo foi aprovado pelo Comitê de Ética em Pesquisa da Universidade Federal de Pernambuco, sob número 13097213.2.0000.5208. O ensaio clínico foi registrado na *International Clinical Trials Registry Platform* (RBR-bjm4) e conduzido no Ambulatório de Doenças Infecciosas / Parasitárias do Hospital das Clínicas da Universidade Federal de Pernambuco / Recife, Brasil.

### Medidas vasculares

Foi utilizado um aparelho de ultrassom General Electric^®^(GE) LOGIQe BT12 DICOM 3.0 AUTO IMT, com transdutor GE 9-L RS Linear, trabalhando na frequência de 7-10 MHz. As medidas foram realizadas de acordo com técnicas padronizadas.^15 , 16^

**Figura 1:**
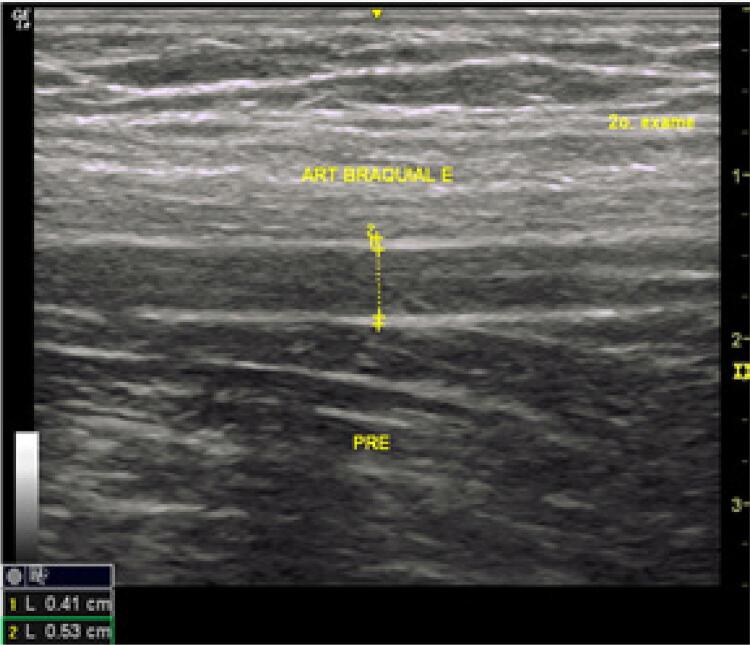
Medida da artéria braquial esquerda antes do estímulo.

DMF: o diâmetro da artéria braquial foi medido em repouso e após estímulo. Para estimular a artéria braquial, um esfigmomanômetro Becton Dickinson^®^ colocado no braço foi inflado a 30 mmHg acima da pressão sistólica por 5 minutos e depois liberado. Um minuto após a liberação do clampeamento, o diâmetro da artéria foi medido novamente. A dilatação normal foi considerada >10% - Figuras 1 e 2 .

**Figura 2:**
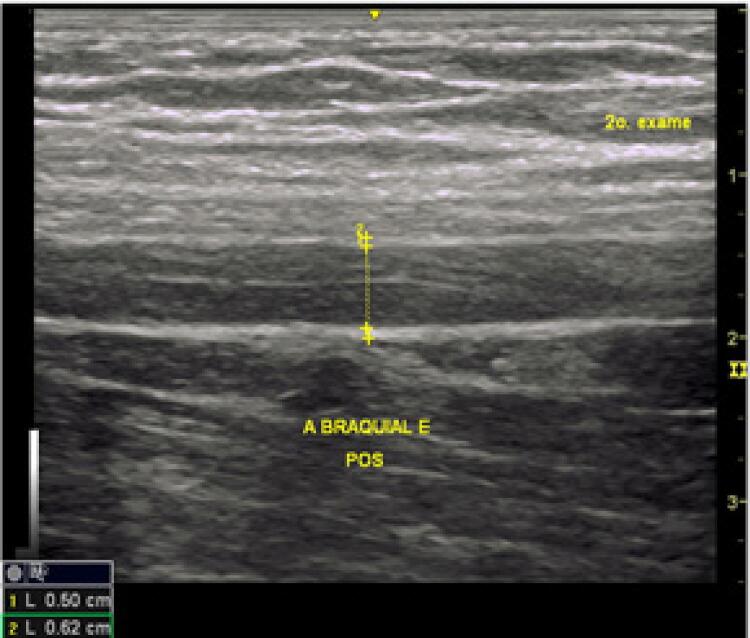
Medida da artéria braquial esquerda após o estímulo.

**Figure 3:**
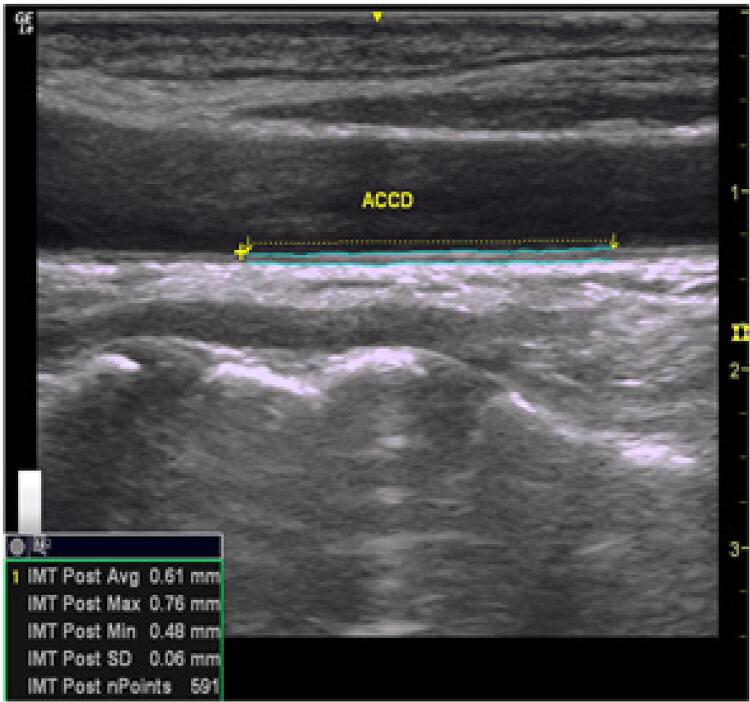
– Medida da espessura médio-intimal da artéria carótida direita.

IMT: A espessura médio-intimal da carótida comum em uma área livre de placas foi considerada uma medida de referência. Foi avaliado nos cortes longitudinal e transversal, do segmento proximal à bifurcação e nas carótidas interna e externa. O IMT foi medido na parede posterior da carótida comum em uma área livre de placas. A placa carotídea foi definida como uma estrutura focal estendendo-se por um mínimo de 0,5 mm até o lúmen do vaso e / ou medindo mais de 50% do valor do IMT adjacente e / ou uma medida de IMT maior que 1,5 mm^17^ ( Figura 3 ).

### Análise estatística

Os dados foram analisados descritivamente pelas estatísticas: média, desvio padrão (média ± DP) ou mediana e intervalo interquartil (IIQ) para as variáveis numéricas e frequências absolutas e percentuais para as variáveis categóricas e foram analisados inferencialmente através de testes estatísticos. Na comparação entre duas categorias, foram utilizados os seguintes testes: teste *t* de Student não-pareado com variâncias iguais ou teste de Mann-Whitney para as variáveis numéricas e teste Qui-quadrado de Pearson ou Exato de Fisher para as variáveis categóricas. O teste *t* de Student foi utilizado com variáveis com distribuição normal e o teste de Mann-Whitney com variáveis que apresentavam distribuição não-normal. O teste Exato de Fisher foi utilizado nos casos em que não foi verificada a condição de uso do teste Qui-quadrado. A verificação da normalidade dos dados foi realizada pelo teste de Shapiro-Wilk e a hipótese de igualdade de variâncias foi verificada pelo teste F de Levene. O nível de significância estatística adotado foi de 5% e os intervalos de confiança foram de 95,0%.

Os dados foram digitados em planilha EXCEL e o programa IBM-SPSS, versão 23, foi utilizado para a realização dos cálculos estatísticos.

## Resultados

As características dos 38 sujeitos incluídos no estudo estão descritas na Tabela 1 . Os resultados demonstraram: média de idade (42,6 anos), tempo de diagnóstico (mediana de 6,5 anos), tempo de terapia antirretroviral (mediana de 6,0 anos). Características da amostra: sexo masculino (52,6%), hipertensos (7,9%), diabéticos (5,3%), tabagistas (15,8%). Algumas características foram descritas por subgrupo, como idade (21-39 e 40-59 anos), etnia (branca, preta e parda) e estado nutricional (peso normal, sobrepeso e obesidade).

**Tabela 1:** – Características clínicas e demográficas dos 38 participantes do estudo

Variável	TOTAL
**Grupo Total:**	**38 (100,0)**
**Idade:** Média ± DP (Mediana)	42,6 ± 8,8 (43,0)
**Faixa etária:** n (%)	
21 a 39	16 (42)
40 a 59	22 (58)
**Sexo:** n (%)	
Masculino	20 (52,6)
Feminino	18 (47,4)
**Etnia:** n (%)	
Branca	15 (39,5)
Preta	4 (10,5)
Parda	19 (50)
**Nível de escolaridade:** n (%)	
Fundamental	13 (34,2)
Médio	18 (47,3)
Superior	7 (18,4)
**IMC:** Mediana (P25;IIQ;P75)	24,2 (21,6; 6,6; 28,2)
**Estado Nutricional:** n (%)	
Peso normal	23 (60,5)
Sobrepeso	8 (21,0)
Obesidade	7 (18,4)
**PAS:** Mediana (P25;IIQ;P75)	120,00 (110,0; 10,0; 120,0)
**PAD:** Mediana (P25;IIQ;P75)	80,00 (70,0; 10,0; 80,0)
HAS: n (%)	
Sim	3 (7,9)
Não	35 (92,1)
**História familiar de doença cardiovascular:** n (%)	
Sim	12 (31,6)
Não	26 (68,4)
**DM:** n (%)	
Sim	2 (5,3)
Não	36 (94,7)
**Fumante:** n (%)	
Sim	6 (15,8)
Não	32 (84,2)
**Tempo desde o diagnóstico:** Mediana (P25;IIQ;P75)	6,50 (4,0; 8,0; 12,0)
**Tempo desde o diagnóstico:** n (%)	
Até 1 ano	4 (10,5)
2 a 5 anos	12 (31,6)
**Continuação**	
6 a 10 anos	12 (31,6)
Acima de 10 anos	10 (26,3)
**Tempo recebendo TARV:** Mediana (P25;IIQ;P75)	6,00 (2,0; 7,8; 9,8)
Até 1 ano: n (%)	5 (13,2)
2 a 5 anos	13 (34,2)
6 a 10 anos	11 (28,9)
Acima de 10 anos	9 (23,7)
**Nadir de células T CD4:** Média ± DP (Mediana)	362,3 ± 239,5 (340,5)
**CD4:** Média ± DP (Mediana)	724,0 ± 354,7 (659,5)
**Regime:** n (%)	
AZT+ 3 TC + EFV	21 (55,3)
TDF + 3TC + EFV	13 (34,2)
AZT + 3TC + NEV	1 (2,6)
NEV + 3TC + TDF	2 (5,26)
AZT + DDI + EFV	1 (2,6)

*Os dados são apresentados como médias, desvio padrão (DP), medianas, intervalo interquartil (IIQ), percentil (P) ou n (%) de indivíduos. IMC:índice de massa corporal; DM: diabetes mellitus; PAS: pressão arterial sistólica; PAD: pressão arterial diastólica; HAS: hipertensão arterial sistêmica; TARV: terapia antirretroviral; AZT: zidovudina; DDI: didanosina; EFV: efavirenz; 3-TC: lamivudina; NVP: nevirapina; TDF: tenofovir.*

### Fatores associados à dilatação mediada por fluxo (DMF) da artéria braquial

Uma diferença estatisticamente significativa foi obtida para a média de idade (p = 0,015). Quando avaliadas as faixas etárias (21-39 anos e 40-59 anos), a significância foi mantida (p = 0,034). Ao avaliar a faixa etária mais avançada, observou-se que houve excelente resposta à dilatação da artéria braquial ( *OR* = 4,37, IC 95%: 1,07 - 17,79), em comparação com a obtida no grupo de 21 a 39 anos.

Quando avaliamos o desfecho em relação ao sexo, obteve-se um resultado limítrofe (p = 0,076, com *OR* = 3,5 (IC 95%: 0,85-14,41) para o sexo feminino. Os demais fatores de risco avaliados não apresentaram significância estatística: hipertensão arterial sistêmica (HAS, p = 0,542); diabetes mellitus (DM; p = 1,00); tabagismo (p = 0,383), como mostrado na Tabela 2 .

**Tabela 2:** – Fatores associados a uma resposta favorável à DMF em 38 pacientes em uso de atorvastatina + aspirina, com baixo risco cardiovascular e carga viral indetectável

	DMF			
Variável	Resposta favorável (Casos)	Sem resposta (Controles)	p-valor	*OR* (IC of 95%)
Grupo Total:	24 (63,2)	14 (36,8)		
**Idade:** Média ± DP (Mediana)	45,3 ± 8,8 (46,0)	38,1 ± 7,2 (36,5)	p^(3)^ = 0,015*	
**Faixa etária:** n (%)			p^(2)^ = 0,034*	
21 a 39 anos	7 (43,8)	9 (56,3)		1,00
40 a 59 anos	17 (77,3)	5 (22,7)		4,37 (1,07-17,79)
**Sexo:** n (%)			p^(2)^ = 0,076	
Masculino	10 (50,0)	10 (50,0)		1,00
Feminino	14 (77,8)	4 (22,2)		3,50 (0,85-14,41)
**Etnia:** n (%)			p^(2)^ = 0,744	
Branca	9 (60,0)	6 (40,0)		1,00
Não-branca	15 (65,2)	8 (34,8)		1,25 (0,33-4,79)
**Nível de escolaridade:** n (%)			p^(4)^ = 0,157	
Fundamental	11 (84,6)	2 (15,4)		**
Médio	9 (50,0)	9 (50,0)		**
Superior	4 (57,1)	3 (42,9)		**
**IMC:** Média ± DP (Mediana)	24,6 ± 4,9 (23,1)	26,5 ± 4,6 (24,9)	p^(3)^ = 0,250	
**Estado nutricional:** n (%)			p^(4)^ = 0,574	
Peso normal	16 (69,6)	7 (30,4)		1,71 (0,30-9,77)
Sobrepeso	4 (50,0)	4 (50,0)		0,75 (0,10-5,77)
Obesidade	4 (57,1)	3 (42,9)		1,00
**PAS:** Mediana (P25;IIQ;P75)	120,0 (110,0;17,5; 127,5)	120,0(110,0;10,0;120,0)	p^(1)^ = 0,747	
**PAD:** Mediana (P25;IIQ;P75)	80,00 (70,0; 10,0; 80,0)	80,00 (70,0; 12,5; 82,5)	p^(1)^ = 0,767	
**HAS:** n (%)			p^(4)^ = 0,542	
Sim	1 (33,3)	2 (66,7)		**
Não	23 (65,7)	12 (34,3)		
**História familiar de doença cardiovascular:** n (%)			p^(4)^ = 1,000	
Sim	8 (66,7)	4 (33,3)		1,25 (0,30-5,26)
Não	16 (61,5)	10 (38,5)		1,00
**DM:** n (%)			p^(4)^ = 1,000	
Sim	1 (50,0)	1 (50,0)		**
Não	23 (63,9)	13 (36,1)		
**Fumante:** n (%)			p^(4)^ = 0,383	
Sim	5 (83,3)	1 (16,7)		**
Não	19 (59,4)	13 (40,6)		
**Tempo desde o diagnóstico:** Média ± DP (Mediana)	8,3 ± 4,8 (8,0)	6,4 ± 5,3 (4,0)	p^(3)^ = 0,264	
**Tempo desde o diagnóstico:** n (%)			p^(2)^ = 0,152	
Até 5 anos	8 (50,0)	8 (50,0)		1,00
6 ou mais anos	16 (72,7)	6 (27,3)		2,67 (0,69-10,36)
**Tempo recebendo TARV:** Mediana (P25;IIQ;P75)	6,50 (3,0; 8,3; 11,3)	3,50 (1,8; 7,2; 9,0)	p^(1)^ = 0,149	
Continuação				
**Tempo recebendo TARV:** n (%)			p^(2)^ = 0,111	
Até 5 anos	9 (50,0)	9 (50,0)		1,00
6 ou mais anos	15 (75,0)	5 (25,0)		3,00 (0,76-11,81)
**Nadir de células T CD4:** Média ± DP (Mediana)	373,8 ± 247,8 (332,5)	342,6 ± 232,3 (354,0)	p^(3)^ = 0,704	
**CD4:** Média ± DP (Mediana)	754,3 ± 391,5 (659,5)	672,1 ± 286,8 (677,5)	p^(3)^ = 0,499	
**Regime:** n (%)			p^(4)^ = 0,724	
AZT + 3TC + EFV	13 (61,9)	8 (38,1)		**
TDF + 3TC + EFV	9 (69,2)	4 (30,8)		**
AZT + 3TC + NEV	-	1 (100,0)		**
NEV + 3TC + TDF	1 (50,0)	1 (50,0)		**
AZT + DDI + EFV	1 (100,0)	-		**

Os dados são apresentados como médias, desvio padrão (DP), medianas, intervalo interquartil (IIQ), percentil (P) ou n (%) de indivíduos. (*) Diferença significativa ao nível de 5,0%. (**) Não foi possível determinar devido à ocorrência de frequências nulas e muito baixas. (1) Usando o teste de Mann-Whitney. (2) Usando o teste Qui-quadrado de Pearson. (3) Usando o teste t de Student com variâncias iguais. (4) Usando o teste exato de Fisher. IMC: índice de massa corporal; DM: diabetes mellitus; PAS: pressão arterial sistólica; PAD: pressão arterial diastólica; HAS: hipertensão arterial sistêmica; TARV: terapia antirretroviral; AZT: zidovudina; DDI: didanosina; EFV: efavirenz; 3-TC: lamivudina; NVP: nevirapina; TDF: tenofovir.

### Fatores associados à redução da espessura médio-intimal (IMT) da carótida

Não foram observadas diferenças estatisticamente significativas para nenhuma das variáveis avaliadas em relação à redução da espessura médio-intimal da carótida: idade (p = 0,706); sexo (p = 0,260), HAS e DM (p = 1,00); tabagismo (p = 0,131), IMC (p = 0,945), como mostrado na Tabela 3 .

**Tabela 3:** – Fatores associados à redução da IMT da carótida em 38 pacientes em uso de atorvastatina + aspirina, com baixo risco cardiovascular e carga viral indetectável

	IMT			
Variável	Redução (Casos)	Sem redução (Controles)	p-valor	*OR* (IC of 95%)
Grupo Total:	29 (76,3)	9 (23,7)		
**Idade:** Média± DP (Mediana)	41,6 ± 8,9 (43,0)	45,9 ± 8,0 (46,0)	p^(1)^ = 0,211	
**Faixa etária:** n (%)			p^(2)^ = 0,706	
21 a 39 anos	13 (81,3)	3 (18,8)		1,63 (0,34-7,79)
40 a 59 anos	16 (72,7)	6 (27,3)		1,00
**Sexo:** n (%)			p^(2)^ = 0,260	
Masculino	17 (85,0)	3 (15,0)		2,83 (0,59-13,63)
Feminino	12 (66,7)	6 (33,3)		1,00
**Etnia:** n (%)			p^(2)^ = 1,000	
Branca	11 (73,3)	4 (26,7)		1,00
Não-branca	18 (78,3)	5 (21,7)	,	1,31 (0,29-5,95)
**Nível de escolaridade:** n (%)			p^(2)^ = 0,782	
Fundamental	9 (69,2)	4 (30,8)		**
Médio	14 (77,8)	4 (22,2)		**
Superior	6 (85,7)	1 (14,3)		**
**IMC:** Mediana (P25;IIQ;P75)	24,20 (21,7; 5,9; 27,6)	23,18 (21,4; 9,3; 30,7)	p^(3)^ = 0,945	
Estado Nutricional:			p^(2)^ = 0,757	
Peso normal	17 (73,9)	6 (26,1)		**
Sobrepeso	7 (87,5)	1 (12,5)		**
Obesidade	5 (71,4)	2 (28,6)		**
**PAS:** Mediana (P25;IIQ;P75)	120,00 (110,0;10,0; 120,0)	120,00 (110,0; 30,0; 140,0)	p^(3)^ = 0,272	
**PAD:** Mediana (P25;IIQ;P75)	80,00 (70,0; 10,0; 80,0)	80,00 (70,0; 15,0; 85,0)	p^(3)^ = 0,653	
Continuação				
HAS: n (%)			p^(2)^ = 1,000	
Sim	3 (100,0)	-		**
Não	26 (74,3)	9 (25,7)		
**História familiar de doença cardiovascular:** n (%)			p^(2)^ = 0,423	
Sim	8 (66,7)	4 (33,3)		1,00
Não	21 (80,8)	5 (19,2)		2,10 (0,45-9,86)
DM: n (%)			p^(2)^ = 1,000	
Sim	2 (100,0)	-		**
Não	27 (75,0)	9 (25,0)		
**Fumante:** n (%)			p^(2)^ = 0,131	
Sim	3 (50,0)	3 (50,0)		1,00
Não	26 (81,3)	6 (18,8)		4,33 (0,70- 27,01)
**Tempo desde o diagnóstico:** Mediana (P25;IIQ;P75)	6,00 (4,0; 8,0; 12,0)	8,00 (3,5; 7,5; 11,0)	p^(3)^ = 0,836	
**Tempo desde o diagnóstico:** n (%)			p^(2)^ = 0,706	
Até 5 anos	13 (81,3)	3 (18,7)		1,63 (0,34-7,79)
6 ou mais anos	16 (72,7)	6 (27,3)		1,00
**Tempo recebendo TARV:** Mediana (P25;IIQ;P75)	6,00 (2,5; 9,5; 12,0)	6,00 (2,0; 6,5; 8,5)	p^(3)^ = 0,593	
**Tempo recebendo TARV:** n (%)			p^(2)^ = 1,000	
Até 5 anos	14 (77,8)	4 (22,2)		1,17 (0,26-5,24)
6 ou mais anos	15 (75,0)	5 (25,0)		1,00
**Nadir de células T CD4:** Média± DP (Mediana)	350,2 ± 236,9 (315,0)	401,3 ± 258,2 (401,0)	p^(1)^ = 0,583	
**CD4:** Média ± DP (Mediana)	750,8 ± 375,6 (677,0)	637,5 ± 277,7 (574,0)	p^(1)^ = 0,410	
**Regime:** n (%)			p^(2)^ = 1,000	
AZT+3TC + EFV	15 (71,4)	6 (28,6)		**
TDF + 3TC + EFV	10 (76,9)	3 (23,1)		**
AZT+3TC + NEV	1 (100,0)	-		**
NEV + 3TC + TDF	2 (100,0)	-		**
AZT + DDI + EFV	1 (100,0)	-		**

Os dados são apresentados como médias, desvio padrão (DP), medianas, intervalo interquartil (IIQ), percentil (P) ou n (%) de indivíduos. (**) Não foi possível determinar devido à ocorrência de frequências nulas e muito baixas. (1) Usando o teste t de Student com variâncias iguais. (2) Usando o teste exato de Fisher. (3) Usando o teste de Mann-Whitney. IMC: índice de massa corporal; DM: diabetes mellitus; PAS: pressão arterial sistólica; PAD: pressão arterial diastólica; HAS: hipertensão arterial sistêmica; TARV: terapia antirretroviral, AZT: zidovudina; DDI: didanosina; EFV: efavirenz; 3-TC: lamivudina; NVP: nevirapina; TDF: tenofovir.

## Discussão

Nosso estudo avaliou pacientes vivendo com HIV, recebendo terapia antirretroviral e com baixo risco cardiovascular, que tomaram a combinação de atorvastatina + aspirina por um período de 6 meses. Realizou-se uma análise exploratória para avaliar os fatores associados à resposta positiva ao tratamento avaliado através das técnicas vasculares de DMF e IMT.

Os resultados demonstraram que indivíduos pertencentes à faixa etária mais avançada (entre 40 e 59 anos) responderam positivamente à combinação atorvastatina + aspirina, ou seja, com aumento da DMF ao final do estudo. Pode-se inferir que indivíduos mais velhos foram expostos por mais tempo à inflamação decorrente do HIV. Sabe-se que existem níveis mais elevados de inflamação nas pessoas com HIV do que nas não infectadas, mesmo aquelas sob controle virológico, e essa exposição é um fator importante na gênese da disfunção endotelial. Esses achados são semelhantes aos obtidos por outros autores, que verificaram que um alto nível de replicação do vírus resulta em piora na dilatação da artéria braquial.^18^ Por outro lado, quanto maior o controle viral, melhor é a função endotelial.^19^ Outra hipótese seria de que indivíduos em uma faixa etária mais avançada estariam mais sujeitos às consequências do processo aterosclerótico relacionado à idade e mais sensíveis aos efeitos deletérios do HIV sobre o endotélio. Por sua vez, nossos achados podem sugerir que esses indivíduos mais idosos seriam mais responsivos às ações pleiotrópicas e anti-inflamatórias da combinação de atorvastatina + aspirina. Nossos achados sugerem que há benefício do uso de estatinas + aspirina como profilaxia primária para doenças cardiovasculares em indivíduos com HIV, que deve ser avaliado de forma diferente em indivíduos de acordo com sua faixa etária, principalmente indivíduos com 40 anos ou mais.^20 , 21^

Quando avaliamos a resposta relacionada ao sexo, obtivemos um resultado limítrofe, no qual o *OR* para o grupo feminino foi igual a 3,5. Embora não tenha apresentado significância estatística, essa resposta chamou nossa atenção, pois sugere que as mulheres podem responder melhor ao tratamento com atorvastatina + aspirina do que os homens. Estudos têm sugerido que, entre as pessoas que vivem com HIV, as mulheres apresentam níveis mais elevados de ativação imunológica e inflamação do que os homens.^22^ Considerando que os medicamentos atualmente em uso têm um efeito importante na redução da inflamação, um mecanismo intrinsecamente relacionado à progressão da aterosclerose, pode-se inferir que esta poderia ser a possível razão para uma resposta mais evidente nas mulheres do que nos homens. Nosso estudo, entretanto, não foi capaz de confirmar essa associação, mas outros que avaliaram um número maior de indivíduos podem ter poder suficiente para alcançar significância estatística. Estudos que associam o sexo com resposta à função endotelial seriam necessários.

Os regimes antirretrovirais utilizados não foram significativamente associados às respostas de DMF e IMT; entretanto, eles incluíram apenas inibidores de transcriptase reversa análogos de nucleosídeos (ITRNs) e não-análogos. Pacientes em uso de inibidores de protease (IP) ou inibidores da integrase (INI) não foram incluídos. Sabe-se que entre os medicamentos utilizados atualmente, os IP causam mais distúrbios metabólicos do que os demais e consequentemente predispõem a um maior risco cardiovascular.^23^ Dube et al.,^24^ em estudo transversal comparando indivíduos com ou sem uso de IP, não observaram diferença na resposta à DMF. No entanto, vários outros autores descobriram maior espessura carotídea medido pelo IMT naqueles em uso de IP em comparação com aqueles que não receberam IP.^25 , 26^

**Figura 4:**
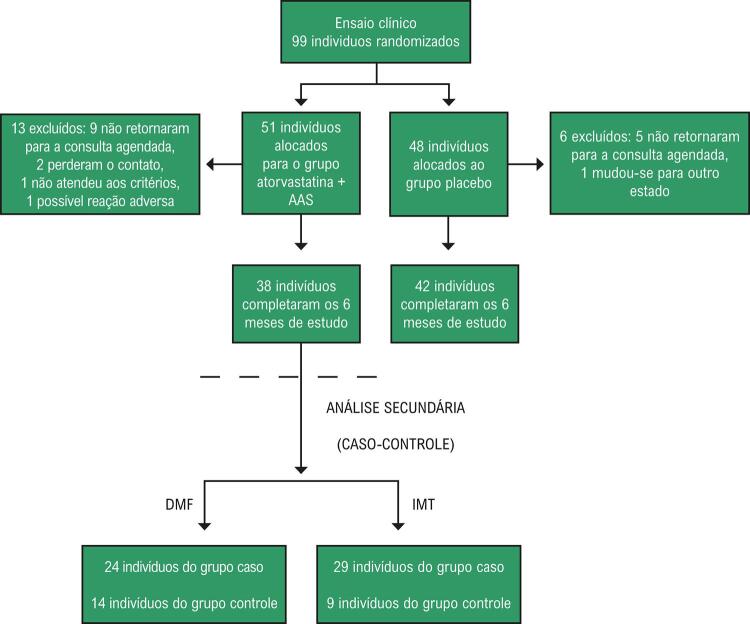
– Fluxograma dos participantes do estudo.

O uso de esquemas de tratamento com grupos restritos de antirretrovirais tem como objetivo homogeneizar os grupos de comparação e evitar que os medicamentos se tornem fatores de confusão quanto à resposta ao uso de atorvastatina + aspirina.

O tabagismo não foi associado às respostas de DMF ou IMT. Deve-se enfatizar que a baixa prevalência do tabagismo pode ter dificultado a avaliação do papel que o mesmo desempenhou. No entanto, deve-se ressaltar que, na avaliação do IMT, os não fumantes apresentaram chance 4,3 vezes maior de obter redução do IMT com atorvastatina + aspirina. Contudo, provavelmente devido ao pequeno número de casos, o intervalo de confiança foi alto (0,70 - 27,01) e não houve significância estatística. Um estudo recente demonstrou que o tabagismo resulta em controle viral e resposta imunológica ruim,^27^ o que, como mencionado anteriormente, resulta em maior risco cardiovascular. Um estudo de coorte relacionou o tabagismo à piora da progressão do espessura carotídea.^28^ Estudos com maior número de pacientes são necessários para determinar o papel dessa intervenção em fumantes.

Nossos achados não revelaram uma associação entre obesidade e resposta da função endotelial medida pela DMF, ou progressão do espessura carotídea (IMT) após receber atorvastatina + aspirina. Um estudo de coorte que monitorou pacientes obesos com HIV e os comparou com obesos não infectados pelo HIV demonstrou uma maior incidência de distúrbios do metabolismo da glicose e inflamação entre aqueles com HIV, embora DMF e IMT não tenham diferido entre os dois grupos.^29^ Dados relataram uma relação entre lipodistrofia e função endotelial deficiente^30^ e aumento da espessura carotídea, principalmente em indivíduos com obesidade visceral.^31^ Em nosso estudo, não diagnosticamos lipodistrofia. Avaliamos apenas a composição corporal com o índice de massa corporal (IMC) e classificamos os indivíduos de acordo com a presença de baixo peso, eutrofia, sobrepeso ou obesidade. Entretanto, como existe uma alta prevalência de lipodistrofia em pacientes com HIV e o IMC não é um índice que possa fornecer uma correlação com esse distúrbio, tal fator de risco deve ser avaliado nesses indivíduos.

A associação da idade com a resposta positiva ao tratamento foi diferente quando comparada aos métodos utilizados para sua avaliação: enquanto a DMF apresentou melhora com o tratamento nos pacientes mais velhos, a avaliação do IMT não demonstrou essa diferença entre os grupos. A DMF e o IMT são frequentemente utilizados como medidas substitutas para aterosclerose subclínica. Enquanto o IMT identifica anormalidades estruturais precoces, a DMF, considerada um bioensaio endotelial, avalia a integridade funcional do vaso.^32^ Há dados que demonstram que os dois métodos são únicos e independentes e não se correlacionam entre si, embora sejam considerados válidos para a detecção da aterosclerose subclínica. Eles provavelmente refletem diferentes aspectos e estágios da aterosclerose inicial.^32 , 33^ Portanto, a divergência dos resultados em nosso estudo está de acordo com a literatura, e demonstra que a DMF mostrou ser capaz de identificar o benefício da utilização da combinação de atorvastatina + aspirina em indivíduos HIV-positivos com idade entre 40-59 anos quando comparados com pacientes mais jovens.

O estudo clínico original demonstrou uma redução percentual dos níveis de LDL nos indivíduos do grupo caso (-19,35%, p = 0,007), mas sem melhora da função endotelial. Consideramos algumas limitações nesse estudo, destacando-se o tempo de uso das estatinas, que foi planejado e realizado por um período de 6 meses. Estudos que demonstraram resultados encorajadores utilizaram as estatinas por períodos muito mais longos do que o nosso, sugerindo um caminho a ser seguido. Outra questão diz respeito ao perfil dos pacientes envolvidos em nosso estudo. Todos apresentavam poucos fatores tradicionais de risco cardiovascular, a carga do HIV estava sob controle e eles estavam em tratamento antirretroviral há vários anos. Essa seleção resultou em um grupo de indivíduos com pouca ou nenhuma inflamação, como demonstrado pelos baixos níveis de marcadores inflamatórios, revelando assim uma população para a qual o uso de estatinas em curto prazo associado à aspirina provavelmente não forneceria resultados eficazes.

Os pontos fortes destacados pelo presente estudo seriam a seleção de indivíduos com baixo risco cardiovascular e o uso de medicamentos antirretrovirais com baixo potencial para causar distúrbios metabólicos. Essas características permitem investigações sobre os possíveis efeitos dos medicamentos e os fatores associados a um melhor desfecho no estágio inicial da doença aterosclerótica, ou seja, o período em que ocorrem alterações no endotélio vascular, sendo, portanto, um processo que pode ser revertido. Uma possível fragilidade, porém, que deve ser destacada, foi o fato de o estudo envolver um número pequeno de indivíduos. Tal amostra pode ter sido insuficiente para detectar possíveis associações a fatores que possivelmente poderiam ser observados em uma maior amostra de indivíduos.

## Conclusões

O estudo revelou que o fator idade influencia a melhora da função endotelial em indivíduos com HIV e baixo risco cardiovascular tratados com a combinação de atorvastatina + aspirina. Também mostrou que a DMF é um método capaz de revelar esse efeito. Estudos semelhantes, envolvendo um maior número de indivíduos, são necessários para confirmar nossa hipótese e apoiar o uso precoce da combinação atorvastatina + aspirina em indivíduos de 40 a 59 anos, em tratamento antirretroviral e com baixo risco cardiovascular para prevenção de doenças cardiovasculares.
