# Supplementary material for: O Efeito da Atorvastatina + Aspirina na Função Endotelial Difere com a Idade em Pacientes com HIV: Um Estudo de Caso-Controle
Source: Arq Bras Cardiol. 2021 Jun 25;117(2):365–75. [Article in Portuguese] doi: 10.36660/abc.20190844 (PMC8395794; doi:10.36660/abc.20190844)
Supplement: Translation

# Translation: The Effect of Atorvastatin + Aspirin on the Endothelial Function Differs with Age in Patients with HIV: A Case-Control Study

**Authors:** Gerson Gomes dos Santos, Paulo Sérgio Ramos Araújo, Kaliene Maria Estevão Leite, Emmanuelle Tenório Godoi, Adriana Ferraz Vasconcelos, Heloisa Ramos Lacerda

**Affiliations:** 1 Brasil Universidade Federal de Pernambuco - Pós-graduação em Medicina Tropical, Recife, PE - Brasil; 2 Brasil Universidade Federal de Alagoas - Instituto de Ciências Farmacêuticas, Maceió, AL - Brasil; 3 Brasil Instituto de Pesquisa Aggeu Magalhães, Recife, PE - Brasil; 4 Brasil Universidade Federal de Pernambuco - Medicina Clínica, Recife, PE - Brasil

**Keywords:** HIV, Carotid Arteries/ultrasonography, Carotid Intima-Media Thickness, Brachial Artery, Atorvastatin, Aspirin, Risk Factors, Endothelium Vascular/physiopathology

## Abstract

**Background:**

Patients with HIV are more likely to present with cardiovascular disease when compared to the general population.

**Objective:**

This was a case-control study that aimed to assess which factors were associated with a reduction in the carotid intima-media thickness (IMT) and an increase in the brachial artery flow-mediated dilation (FMD) in HIV patients who received atorvastatin + aspirin during a period of 6 months.

**Methods:**

A secondary analysis of a clinical trial was conducted, which included people living with HIV infection and low cardiovascular risk. A total of 38 patients allocated to the intervention arm and treated for 6 months with a combination of atorvastatin + aspirin were included. All participants underwent a carotid and brachial artery ultrasound, both at the beginning and the end of the study. Cases that responded with an increase of >10% of the brachial dilatation (FMD) and reduction of the carotid intima-media thickness (IMT) were considered cases, and those who did not respond were considered controls. We assessed the factors associated with the positive responses obtained through IMT and FMD.

**Results:**

A reduction in the IMT was not significantly associated with any of the evaluated risk factors: age (p=0.211), gender (p=0.260), smoking (p=0.131) or time since HIV diagnosis (p=0.836). An increase in the FMD was significantly associated with age amongst those in the 40-59 age group, p = 0.015 (OR = 4.37; 95% CI: 1.07-17.79).

**Conclusions:**

Older individuals were more likely to present with an increased FMD after 6 months of treatment with atorvastatin + aspirin.

## Introduction

Life expectation and quality of life among people infected with HIV has increased significantly over recent decades. This is due to the great success of antiretroviral therapy.^1^ Living with the virus has now become a chronic condition, which imposes the challenge of maintaining viral suppression coupled with the management of age-related comorbidities.^2^ A substantial increase in non-AIDS-related deaths, such as those related to cardiovascular diseases, has been reported,^3^ and are more prevalent in these individuals, when compared to the general population.^4 , 5^

An early marker for atherosclerosis is endothelial dysfunction and preventing this dysfunction may be an alternative for preventing future cardiovascular events. Aspirin and, more recently, statins have demonstrated pleiotropic effects, such as: immunomodulatory, and antithrombogenic and anti-inflammatory effects. Such medications may be an alternative for the primary and secondary prevention of these events among people living with HIV.^6 - 8^

Observational and interventional studies have evaluated the effects of statins in improving endothelial function, and the progression of carotid thickening in individuals both with and without HIV. These studies have used non-invasive ultrasound techniques, such as FMD, which measures the mediated flow of the brachial artery, and IMT, which measures carotid intima-media thickening, and have reported conflicting results.^9 - 12^ To contribute to this discussion, our study aims to assess the factors associated with endothelial function improvement and carotid thickness measured by FMD and IMT in subjects with HIV, with a viral load under control, who were treated with a combination of atorvastatin + aspirin for a period of 6 months.

## Methods

This was a secondary analysis of a clinical trial not yet published^13^ in which 80 participants who presented with low cardiovascular risk, as measured by the Framingham Risk Score (FRS), and an undetectable viral load were assessed.

The study was planned for 6 months, using 2 nucleoside reverse transcriptase inhibitors and 1 non-nucleoside inhibitor regimens, which were randomized into intervention and placebo groups. Thirty-eight participants were allocated to the intervention group and treated for 6 months with a combination of 20mg atorvastatin + 100mg aspirin, and 42 received placebo. The study assessed the efficacy of the drug combination through ultrasound measurements of the increased brachial artery dilation (FMD), reduced carotid thickening (IMT), and inflammatory markers (ultrasensitive-PCR, ICAM-1, VCAM-1, IL-1, IL-6, TNF-α) and no difference was found between the intervention group and the placebo group.

In the case-control study presented herein, 38 individuals from the intervention group of the aforementioned clinical trial were included. The aim was to assess subgroups that could benefit from the use of atorvastatin 20mg and aspirin 100mg in reducing subclinical atherosclerosis and cardiovascular disease.

In the first part of the case-control study, a total of 38 individuals were divided into 24 cases, which were those who had a favorable response in FMD (≥10% of brachial artery dilation according to the method described by Regattieri et al.^14^ and 14 patients who were considered controls, as they did not show response in FMD.

In the second part of the case-control study, the 38 subjects were divided into 29 cases, which were the individuals who showed a reduction in the carotid IMT, and 9 controls who did not show a reduction in the carotid IMT.

All individuals signed the free and informed consent form. The study was approved by the Research Ethics Committee of Universidade Federal de Pernambuco, under number 13097213.2.0000.5208. The clinical trial was registered at the International Clinical Trials Registry Platform (RBR-bjm4) and conducted at the Infectious/Parasitic Diseases Outpatient Clinic at Hospital das Clínicas, Universidade Federal de Pernambuco/Recife, Brazil.

### Vascular measurements

A General Electric™ (GE) LOGIQe BT12 DICOM 3.0 AUTO IMT ultrasound device was used, with a GE 9-L RS Linear transducer, working at a frequency of 7-10 MHz. The measurements were performed according to standardized techniques.^15 , 16^

FMD: The brachial artery diameter was measured at rest and after stimulation. To stimulate the brachial artery, a Becton Dickinson™ sphygmomanometer placed on the arm was inflated to 30mmHg above the systolic pressure for 5 minutes, and then released. One minute after releasing the clamp, the diameter of the artery was measured once again. Normal dilation was considered > 10% - Figures 1 and 2 .

**Figure 1:** Measurement of the left brachial artery before the stimulus.

**Figure 2:** Medida da artéria braquial esquerda após o estímulo.

IMT: The common carotid intima-media thickness in a plaque-free area was considered a reference measure. It was assessed in the longitudinal and cross-sectional sections, from the proximal segment to the bifurcation and the internal and external carotids. The IMT was measured on the posterior wall of the common carotid in a plaque-free area. The carotid plaque was defined as a focal structure extending for a minimum of 0.5 mm to the lumen of the vessel and/or measuring more than 50% of the adjacent IMT value and/or an IMT measurement greater than 1.5 mm^17^ ( Figure 3 ).

**Figure 3:** – Mean intimal thickness measurement of the right carotid artery.

### Statistical analysis

The data were descriptively analyzed through the statistics: mean, standard deviation (mean ± SD) or median and interquartile range (IQR) for numerical variables and absolute and percentage frequencies for categorical variables and were analyzed inferentially through statistical tests. In the comparison between two categories, the following tests were used: unpaired Student’s t-test with equal variances or Mann-Whitney test for the numerical variables and Pearson’s Chi-square test or Fisher’s Exact for the categorical variables. Student’s t-test was used with variables with normal distribution and Mann-Whitney’s test with variables with a non-normal distribution. Fisher’s Exact test was used in cases where the condition for using the Chi-square test was not verified. The verification of data normality was performed by the Shapiro-Wilk’s test and the hypothesis of equality of variances through the Levene F-test. The level of statistical significance adopted was 5% and the confidence intervals were 95.0%

The data were entered into the EXCEL spreadsheet and the IMB-SPSS program, version 23, was used to perform the statistical calculations.

## Results

The characteristics of the 38 subjects included in the study are described in Table 1 . The results demonstrated: mean age (42.6 years), time since diagnosis (median – 6.5 years), antiretroviral therapy time (median - 6.0 years). Characteristics of the sample: male gender (52.6%), hypertensive (7.9%), diabetics (5.3%), smokers (15.8%). Some characteristics were described by subgroup, such as age (21-39 and 40-59 years), ethnicity (white, black and brown), and nutritional status (ideal weight, overweight and obesity).

**Table 1:** – Clinical and demographic characteristics of the 38 study participants

Variable	TOTAL
**Total Group:**	**38 (100,0)**
**Age:** Mean ± SD (Median)	42.6 ± 8.8 (43.0)
**Age range:** n (%)	
21 to 39	16 (42)
40 to 59	22 (58)
**Gender:** n (%)	
Male	20 (52.6)
Female	18 (47.4)
**Ethnicity:** n (%)	
White	15 (39.5)
Black	4 (10.5)
Brown	19 (50)
**Level of education:** n (%)	
Primary Education	13 (34.2)
Secondary Education	18 (47.3)
Higher Education	7 (18.4)
**BMI:** Median (P25;IR;P75)	24.2 (21.6; 6.6; 28.2)
**Nutritional status:** n (%)	
Ideal weight	23 (60.5)
Overweight	8 (21.0)
Obese	7 (18.4)
**SBP:** Median (P25;IR;P75)	120.00 (110.0; 10.0; 120.0)
**DBP:** Median (P25;IR;P75)	80.00 (70.0; 10.0; 80.0)
SAH: n (%)	
Yes	3 (7.9)
No	35 (92.1)
**Family history of cardiovascular disease:** n (%)	
Yes	12 (31.6)
No	26 (68.4)
**DM:** n (%)	
Yes	2 (5.3)
No	36 (94.7)
**Smoker:** n (%)	
Yes	6 (15.8)
No	32 (84.2)
**Time since diagnosis:** Median (P25;IQR;P75)	6.50 (4.0; 8.0; 12.0)
**Time since diagnosis:** n (%)	
Up to 1 year	4 (10.5)
2 to 5	12 (31.6)
6 to 10	12 (31.6)
Over 10	10 (26.3)
**Continuation**	
**Time on ART:** Median (P25;IQR;P75)	6.00 (2.0; 7.8; 9.8)
Up to 1 year: n (%)	5 (13.2)
2 to 5	13 (34.2)
6 to 10	11 (28.9)
Over 10	9 (23.7)
**NadirTCD4:** Mean ± SD (Median)	362.3 ± 239.5 (340.5)
**CD4:** Mean ± SD (Median)	724.0 ± 354.7 (659.5)
**Regimen:** n (%)	
AZT+ 3 TC + EFV	21 (55.3)
TDF + 3TC + EFV	13 (34.2)
AZT + 3TC + NEV	1 (2.6)
NEV + 3TC + TDF	2 (5.26)
AZT + DDI + EFV	1 (2.6)

*Data are presented as means, standard deviation (SD), medians, Interquartile Range (IQR), percentile (P) or n (%) of individuals. BMI: body mass index; DM: diabetes mellitus; SBP: systolic blood pressure; DBP: diastolic blood pressure; SAH: systemic arterial hypertension; ART: antiretroviral therapy; AZT: zidovudine; DDI: didanosine; EFV: efavirenz; 3-TC: lamivudine; NVP: nevirapine; TDF: tenofovir.*

### Factors associated with brachial artery flow-mediated dilation (FMD)

A statistically significant difference was obtained for the mean age (p = 0.015). When age ranges were assessed (21-39 years and 40-59 years), the significance was maintained (p = 0.034). When assessing the older age group, it was observed that there was an excellent response to brachial artery dilation (OR=4.37, CI 95%: 1.07 - 17.79), compared to that obtained in the 21- 39 year-old group.

When we assessed the outcome regarding sex, a borderline result was obtained (p = 0.076, with an OR = 3.5 (CI 95%: 0.85-14.41) for female subjects. The other risk factors assessed did not show any statistical significance: systemic arterial hypertension (SAH, p = 0.542); diabetes mellitus (DM; p = 1.00); smoking (p = 0.383) in Table 2 .

**Table 2:** – Factors associated with a favorable response to FMD amongst 38 patients receiving atorvastatin + aspirin, with low cardiovascular risk and an undetectable viral load

	FMD			
Variable	Favorable response (Cases)	No response (Controls)	p-value	OR (95%CI)
Total Group:	24 (63.2)	14 (36.8)		
**Age:** Mean ± SD (Median)	45.3 ± 8.8 (46.0)	38.1 ± 7.2 (36,5)	p^(3)^= 0.015*	
**Age Range:** n (%)			p^(2)^= 0.034*	
21 to 39	7 (43.8)	9 (56.3)		1.00
40 to 59	17 (77.3)	5 (22.7)		4.37 (1.07-17.79)
**Gender:** n (%)			p^(2)^= 0.076	
Male	10 (50.0)	10 (50.0)		1.00
Female	14 (77.8)	4 (22.2)		3.50 (0.85-14.41)
**Ethnicity:** n (%)			p^(2)^= 0.744	
White	9 (60.0)	6 (40.0)		1.00
Non-white	15 (65.2)	8 (34.8)		1.25 (0.33-4.79)
**Level of education:** n (%)			p^(4)^= 0.157	
Primary Education	11 (84.6)	2 (15.4)		**
Secondary Education	9 (50.0)	9 (50.0)		**
Higher Education	4 (57.1)	3 (42.9)		**
**BMI:** Mean ± SD (Median)	24.6 ± 4.9 (23,1)	26.5 ± 4.6 (24,9)	p^(3)^= 0.250	
**Nutritional status:** n (%)			p^(4)^= 0.574	
Ideal weight	16 (69.6)	7 (30.4)		1.71 (0.30-9.77)
Overweight	4 (50.0)	4 (50.0)		0.75 (0.10-5.77)
Obese	4 (57.1)	3 (42.9)		1.00
**SBP:** Median (P25;IQR;P75)	120,0 (110,0;17,5; 127,5)	120,0(110,0;10,0;120,0)	p^(1)^= 0.747	
**DBP:** Median (P25;IQR;P75)	80,00 (70,0; 10,0; 80,0)	80,00 (70,0; 12,5; 82,5)	p^(1)^= 0.767	
**SAH:** n (%)			p^(4)^= 0.542	
Yes	1 (33.3)	2 (66.7)		**
No	23 (65.7)	12 (34.3)		
**Family history of cardiovascular disease:** n (%)			p^(4)^= 1.000	
Yes	8 (66.7)	4 (33.3)		1.25 (0.30-5.26)
No	16 (61.5)	10 (38.5)		1.00
**DM:** n (%)			p^(4)^= 1.000	
Yes	1 (50.0)	1 (50.0)		**
No	23 (63.9)	13 (36.1)		
**Smoker:** n (%)			p^(4)^= 0.383	
Yes	5 (83.3)	1 (16.7)		**
No	19 (59.4)	13 (40.6)		
**Time since diagnosis:** Mean ± SD (Median)	8.3 ± 4.8 (8.0)	6.4 ± 5.3 (4.0)	p^(3)^= 0.264	
**Time since diagnosis:** n (%)			p^(2)^= 0.152	
Up to 5 years	8 (50.0)	8 (50.0)		1.00
6 or more years	16 (72.7)	6 (27.3)		2.67 (0.69-10.36)
**Time on ART:** Median (P25;IQR;P75)	6.50 (3.0; 8.3; 11.3)	3.50 (1.8; 7.2; 9.0)	p^(1)^= 0.149	
Continuation				
**Time on ART:** n (%)			p^(2)^= 0.111	
Up to 5 years	9 (50.0)	9 (50.0)		1.00
6 or more years	15 (75.0)	5 (25.0)		3.00 (0.76-11.81)
**NadirTCD4:** Mean ± SD (Median)	373.8 ± 247.8 (332,5)	342.6 ± 232.3 (354.0)	p^(3)^= 0.704	
**CD4:** Mean ± SD (Median)	754.3 ± 391.5 (659.5)	672.1 ± 286.8 (677.5)	p^(3)^= 0.499	
**Regimen:** n (%)			p^(4)^= 0.724	
AZT + 3TC + EFV	13 (61.9)	8 (38.1)		**
TDF + 3TC + EFV	9 (69.2)	4 (30.8)		**
AZT + 3TC + NEV	-	1 (100.0)		**
NEV + 3TC + TDF	1 (50.0)	1 (50.0)		**
AZT + DDI + EFV	1 (100.0)	-		**

Data are presented as means, standard deviation (SD), medians, Interquartile Range (IQR), percentile (P) or n (%) of individuals. (*) Significant difference at the level of 5.0%. (**) This could not be determined due to the occurrence of null and very low frequencies. (1) Using Mann-Whitney test. (2) Using Pearson’s Chi-square test. (3) Using the Student’s t test with equal variances. (4) Using Fisher’s exact test. BMI: body mass index; DM: diabetes mellitus; SBP: systolic blood pressure; DBP: diastolic blood pressure; SAH: systemic arterial hypertension; ART: antiretroviral therapy; AZT: zidovudine; DDI, didanosine; EFV, efavirenz; 3-TC, lamivudine; NVP, nevirapine; TDF, tenofovir.

### Factors associated with a reduction in the carotid intima-media thickness (IMT)

No statistically significant differences were observed for any of the variables assessed in relation to a reduction in the carotid intima-media thickness: age (p = 0.706); gender (p = 0.260), SAH and DM (p = 1.00); smoking (p = 0.131), BMI (p = 0.945), as shown in Table 3 .

**Table 3:** – Factors associated with a reduction in carotid IMT amongst 38 patients receiving atorvastatin + aspirin, with low cardiovascular risk and an undetectable viral load

	IMT			
Variable	Reduction (Cases)	No reduction (Controls)	p-value	OR (95% CI) )
Total Group:	29 (76.3)	9 (23.7)		
**Age:** Mean ± SD (Median)	41.6 ± 8.9 (43.0)	45.9 ± 8.0 (46.0)	p^(1)^= 0.211	
**Age range:** n (%)			p^(2)^= 0.706	
21 to 39	13 (81.3)	3 (18.8)		1.63 (0.34-7.79)
40 to 59	16 (72.7)	6 (27.3)		1.00
**Gender:** n (%)			p^(2)^= 0.260	
Male	17 (85.0)	3 (15.0)		2.83 (0.59-13.63)
Female	12 (66.7)	6 (33.3)		1.00
**Ethnicity:** n (%)			p^(2)^= 1.000	
White	11 (73.3)	4 (26.7)		1.00
Non-white	18 (78.3)	5 (21.7)	.	1.31 (0.29-5.95)
**Level of education:** n (%)			p^(2)^= 0.782	
Primary Education	9 (69.2)	4 (30.8)		**
Secondary Education	14 (77.8)	4 (22.2)		**
Higher Education	6 (85.7)	1 (14.3)		**
**BMI:** Median (P25;IQR;P75)	24.20 (21.7; 5.9; 27.6)	23.18 (21.4; 9.3; 30.7)	p^(3)^= 0.945	
Nutritional status:			p^(2)^= 0.757	
Ideal weight	17 (73.9)	6 (26.1)		**
Overweight	7 (87.5)	1 (12.5)		**
Obese	5 (71.4)	2 (28.6)		**
**SBP:** Median (P25;IQR;P75)	120.00 (110.0;10.0; 120.0)	120.00 (110.0; 30.0; 140.0)	p^(3)^= 0.272	
**DBP:** Median (P25;IQR;P75)	80.00 (70.0; 10.0; 80.0)	80.00 (70.0; 15.0; 85.0)	p^(3)^= 0.653	
Continuation				
SAH: n (%)			p^(2)^= 1.000	
Yes	3 (100.0)	-		**
No	26 (74.3)	9 (25.7)		
**Family history of cardiovascular disease:** n (%)			p^(2)^= 0.423	
Yes	8 (66.7)	4 (33.3)		1.00
No	21 (80.8)	5 (19.2)		2.10 (0.45-9.86)
DM: n (%)			p^(2)^= 1.000	
Yes	2 (100.0)	-		**
No	27 (75.0)	9 (25.0)		
**Smoker:** n (%)			p^(2)^= 0.131	
Yes	3 (50.0)	3 (50.0)		1.00
No	26 (81.3)	6 (18.8)		4.33 (0.70- 27.01)
**Time since diagnosis:** Median (P25;IQR;P75)	6.00 (4.0; 8.0; 12.0)	8.00 (3.5; 7.5; 11.0)	p^(3)^= 0.836	
**Time since diagnosis:** n (%)			p^(2)^= 0.706	
Up to 5 years	13 (81.3)	3 (18.7)		1.63 (0.34-7.79)
6 or more years	16 (72.7)	6 (27.3)		1.00
**Time on ART:** Median (P25;IQR;P75)	6.00 (2.5; 9.5; 12.0)	6.00 (2.0; 6.5; 8.5)	p^(3)^= 0.593	
**Time on ART:** n (%)			p^(2)^= 1.000	
Up to 5 years	14 (77.8)	4 (22.2)		1.17 (0.26-5.24)
6 or more years	15 (75.0)	5 (25.0)		1.00
**NadirTCD4:** Mean ± SD (Median)	350.2 ± 236.9 (315.0)	401.3 ± 258.2 (401.0)	p^(1)^= 0.583	
**CD4:** Mean ± SD (Median)	750.8 ± 375.6 (677.0)	637.5 ± 277.7 (574.0)	p^(1)^= 0.410	
**Regimen:** n (%)			p^(2)^= 1.000	
AZT+3TC + EFV	15 (71.4)	6 (28.6)		**
TDF + 3TC + EFV	10 (76.9)	3 (23.1)		**
AZT+3TC + NEV	1 (100.0)	-		**
NEV + 3TC + TDF	2 (100.0)	-		**
AZT + DDI + EFV	1 (100.0)	-		**

Data are presented as means, standard deviation (SD), medians, Interquartile Range (IQR), percentile (P) or n (%) of individuals. (**) This could not be determined due to the occurrence of null and very low frequencies. (1) Using the Student’s t test with equal variances. (2) Using Fisher’s exact test. (3) Using Mann-Whitney test. BMI, body mass index; DM: diabetes mellitus; SBP: systolic blood pressure; DBP: diastolic blood pressure; SAH: systemic arterial hypertension; ART: antiretroviral therapy; AZT: zidovudine; DDI: didanosine; EFV: efavirenz; 3-TC: lamivudine; NVP: nevirapine; TDF: tenofovir.

## Discussion

Our study assessed patients living with HIV, receiving antiretroviral therapy and with a low cardiovascular risk, who took a combination of atorvastatin + aspirin during a period of 6 months. An exploratory analysis was performed in order to evaluate the factors associated with a positive response to the treatment assessed through FMD and IMT vascular techniques.

The results have demonstrated that individuals belonging to the older age group (between 40 and 59 years) responded positively to a combination of atorvastatin + aspirin, i.e., with increased FMD by the end of the study. It may be inferred that older individuals have been exposed for a longer period to the inflammation resulting from the HIV. It is known that there are higher levels of inflammation in people with HIV than in non-infected people, even those under virological control, and this exposure is an important factor in the genesis of endothelial dysfunction. These findings are similar to those obtained by other authors, who have verified that a high level of virus replication results in a brachial artery dilation worsening.^18^ Conversely, the higher the viral control, the better the endothelial function.^19^ Another hypothesis would be that individuals at an older age range would be more prone to the consequences of the age-related atherosclerotic process and more sensitive to the deleterious effects of HIV on the endothelium. In turn, our findings may suggest that these older individuals would be more responsive to the pleiotropic and anti-inflammatory actions of the combination of atorvastatin + aspirin. Our findings suggest that there is a benefit of the use of statins + aspirin as a primary prophylaxis for cardiovascular disease in individuals with HIV, which should be assessed differently in individuals according to their age group, particularly individuals aged 40 years or older.^20 , 21^

When we assessed the response related to gender, we obtained a borderline result, in which the OR for the female group was equal to 3.5. Although there was no statistical significance, this response nonetheless attracted our attention, since it suggests that females may respond better to treatment with atorvastatin + aspirin than males. Studies have suggested that amongst people living with HIV, women show higher levels of immune activation and inflammation than men.^22^ Considering that the currently used medications have an important effect in reducing inflammation, a mechanism intrinsically related to the progression of atherosclerosis, one could infer that this may be the possible reason for a more evident response in women than in men. Our study, however, was unable to confirm this association, but others that have assessed a larger number of individuals may have sufficient power to obtain statistical significance. Studies that associate gender with response to endothelial function would be necessary.

The antiretroviral regimens used were not significantly associated with FMD and IMT responses; however, they only included analogue and non-analogue nucleoside NRTIs. Patients receiving protease inhibitors (PIs) or integrase inhibitors (INI) were not included. It is known that amongst the currently used medications, the PIs cause more metabolic disorders than the others and, consequently, they predispose to a higher cardiovascular risk.^23^ Dube et al.,^24^ in a cross-sectional study comparing individuals with or without the use of PIs, observed no difference regarding the response to FMD. However, several other authors have discovered greater carotid thickening measured by IMT in those receiving PIs when compared to those not receiving them.^25 , 26^

The use of regimens with restricted groups of antiretroviral drugs have aimed to homogenize the comparison groups and prevent medications from becoming confounding factors regarding the response to atorvastatin + aspirin.

Smoking was not associated with FMD or IMT responses. It should be emphasized that the low prevalence of smoking may have made it difficult to assess the role it played. However, it should be noted that in the IMT assessment, non-smokers showed a 4.3-fold higher chance of obtaining an IMT reduction with atorvastatin + aspirin. However, probably due to the small number of cases, the confidence interval was high (0.70 - 27.01) and there was no statistical significance. One recent study demonstrated that smoking results in poor viral control and immune response,^27^ which, as previously mentioned, results in a higher cardiovascular risk. One cohort study related smoking to a worsening progression of carotid thickening.^28^ Studies with a higher number of patients are necessary in order to determine the role of this intervention in smokers.

Our findings revealed no association between obesity and an endothelial function response measured by FMD, or carotid thickness (IMT) progression after receiving atorvastatin + aspirin. A cohort study that monitored obese patients with HIV and compared them with non-HIV-infected obese individuals, demonstrated a higher incidence of glucose metabolism disorders and inflammation amongst those with HIV, although FMD and IMT did not differ between the two groups.^29^ Data have reported a relationship between lipodystrophy and poor endothelial function^30^ and increased carotid thickening, especially among individuals with visceral obesity.^31^ In our study, we did not diagnose lipodystrophy. We only assessed body composition with the body mass index (BMI) and classified individuals according to low weight, normal weight, overweight or obesity. However, because there is a high prevalence of lipodystrophy among HIV patients, and BMI is not an index that may provide us with a correlation with this disorder, this risk factor should be evaluated in these individuals.

The association of age with a positive response to treatment was different when compared to the methods used for its assessment: while FMD displayed an improvement with treatment in the older patients, the IMT assessment did not demonstrate this difference between the groups. FMD and IMT are frequently used as surrogate measures for subclinical atherosclerosis. While IMT identifies early structural abnormalities, FMD, considered an endothelial bioassay, assesses the functional integrity of the vessel.^32^ There are data demonstrating that the two methods are unique and independent and do not correlate with one other, although they are considered valid for detecting subclinical atherosclerosis. They probably reflect different aspects and stages of early atherosclerosis.^32 , 33^ Therefore, the divergence of the results in our study is consistent with the literature and demonstrates that FMD has shown to be able to identify the benefit of using the combination of atorvastatin + aspirin in HIV-positive individuals aged 40-59 years when compared to younger patients.

**Figure 4:** – Flow chart of the study participants.

The original clinical study demonstrated a percentage reduction in LDL levels in individuals in the case group (-19.35%, p= 0.007), but without improving endothelial function. We considered some limitations in that study, and we would highlight the time receiving statins, which was planned and conducted for a duration of 6 months. Studies that have demonstrated encouraging results used statins for much longer periods than ours, thereby suggesting a path to be followed. One further question concerns the profile of the patients involved in our study. They all presented with few traditional factors of cardiovascular risk, the HIV viral load was well under control and they had been on antiretroviral treatment for several years. This selection resulted in a group of individuals with little or no inflammation, as shown by the low levels of inflammatory markers, thus revealing a population for which the short-term use of statins associated with aspirin would probably not provide any effective results.

The strong points highlighted by the present study would be the selection of individuals with low cardiovascular risk and the use of antiretroviral drugs with a low potential for causing metabolic disorders. These characteristics enable investigations into the possible effects of the drugs and the factors associated with a better outcome in an early stage of atherosclerotic disease, i.e., the period in which changes in the vascular endothelium occur, being therefore a process that can be reversed. One possible weak point, however, which should be highlighted, was the fact that the study involved a small number of individuals. The present sample may have been insufficient to detect possible associations to factors that could possibly be observed in a larger sample of individuals.

## Conclusions

The study has shown that the age factor influences endothelial function improvement in subjects with HIV and low cardiovascular risk receiving a combination of atorvastatin + aspirin. It has also shown that FMD is a method capable of disclosing this effect. Similar studies involving a greater number of individuals are needed to confirm our hypothesis and to support the early use of the combination of atorvastatin + aspirin in subjects aged 40 to 59 years, undergoing antiretroviral treatment and with low cardiovascular risk for the prevention of cardiovascular disease.
